# Diffusive Memristive Switching on the Nanoscale, from Individual Nanoparticles towards Scalable Nanocomposite Devices

**DOI:** 10.1038/s41598-019-53720-2

**Published:** 2019-11-22

**Authors:** Alexander Vahl, Niko Carstens, Thomas Strunskus, Franz Faupel, Abdou Hassanien

**Affiliations:** 1 Institute for Materials Science – Chair for Multicomponent Materials, Faculty of Engineering, Christian-Albrechts-University of Kiel, Kaiserstraße 2, D-24143 Kiel, Germany; 2 Department of Condensed Matter Physics, J. Stefan Institute, Jamova 39, 1000 Ljubljana, Slovenia

**Keywords:** Electronic devices, Electronic properties and materials, Nanoparticles

## Abstract

Nanoscale memristive phenomena are of great interest not only to miniaturize devices and improve their performance but also to understand the details of the underlying mechanism. Herein, we utilize conductive atomic force microscopy (C-AFM) as a non-invasive method to examine the nanoscale memristive properties of individual noble metal alloy nanoparticles that are sparsely encapsulated in a thin SiO_2_ dielectric matrix. The measurement of current-voltage hysteresis loops at the level of individual nanoparticles, enabled by the nanoscopic contact area of the C-AFM tip, indicates reliable memristive switching for several hours of continuous operations. Alongside the electrical characterization on the nanoscale, the method of C-AFM offers the potential for *in situ* monitoring of long term operation induced morphological alterations and device failure, which is demonstrated at the example of nanoparticle-based devices with additional Cr wetting layer. The application of alloy nanoparticles as reservoir for mobile silver species effectively limits the formation of stable metallic filaments and results in reproducible diffusive switching characteristics. Notably, similar behaviour is encountered on macroscopic nanocomposite devices, which incorporate multiple stacks of nanoparticles and offer a high design versatility to tune switching properties and engineer scalable memristive devices with diffusive switching characteristics. No additional forming step is required for the operation of the presented alloy nanoparticle based memristive devices, which renders them very attractive for applications.

## Introduction

With the postulation of the experimental realization of a memristor in 2008^1^, Strukov *et al*. related resistive switching phenomena in sub-stoichiometric oxide thin films to the theoretical model of a memristor as initially proposed by L. Chua in 1971^2^, and initiated a huge increase in research interest in this field. While the memristor as a fundamental element has been under severe debate recently^3^, the innovative power of the use of memristive switching phenomena remains unquestioned. Within the past decade, a broad variety of device concepts was reported in the context of memristive switching, which range from electro-chemical metallization (ECM) over valance change mechanism (VCM) and phase change materials (PCM) and beyond^4–7^. Memristive devices are commonly discussed as promising devices for an application as novel memory^8^, for beyond-von-Neumann logics^9^ and in the context of neuromorphic engineering^10–14^. Among the various reported devices, different switching characteristics are commonly observed, including bipolar, unipolar and diffusive memristive switching^4,15,16^. While in the context of the application as memory device typically bipolar and unipolar switching characteristics are preferred, diffusive memristive devices offer the potential of being used as selector devices or as true random number generators^17,18^.

Amongst the various device classes, special interest is paid to memristive devices relying on ECM, i.e. the reversible formation of a conductive path upon field-driven motion of mobile metal cations (e.g. silver cations) between two electrodes. Typical setup of such devices consists of a dielectric layer, which is sandwiched between two planar electrodes of which one serves as a reservoir for mobile metallic cations (e.g. Cu or Ag). The underlying mechanisms that lead to the reversible changes in resistivity trace back to local rearrangement of atoms and ions on the nanoscale, even in macroscopic memristive devices^19^. As such, recently there is a high interest to progress from planar electrodes towards nanoscale or nanostructured electrodes. In this context, inert Pt nanoparticles dispersed in SiO_2_ matrix are discussed as efficient means to predefine switching channels and locally increase electrical fields and resistive switching is reported in networks of Au nanoparticles^20,21^. For an optimization of switching uniformity in ECM memristive devices, nanostructured electrodes (e.g. nanocones) have been successfully prepared and quantum-dot electrodes have been applied for well-defined local field enhancement at the nanoscale^22–25^. Instead of nanostructured bulk electrodes, also nanoparticles as reservoirs for the mobile metallic species are under investigation, e.g., Ag nanoparticles embedded in dielectric matrices such as a-Si, SiO_2_, TiO_2_, HfO_2_ or MgO^15,18,26,27^. This transition towards nanoparticles for mediating memristive switching is a consequent step as it combines the advantages of local enhancement of the electrical field by the nanoparticles in the dielectric matrix and the pre-definition of the location of memristive switching.

In this work, we substantially extend the concept of nanoparticle-based memristive switching by using gas phase synthesis of alloy nanoparticles and sequential deposition to prepare nanoparticles with controlled size, composition and coverage and embed them into a SiO_2_ matrix in a controlled manner. Compared to earlier studies in this field, in which nanoparticles were mainly formed by self-organization in a co-deposition process, the gas phase synthesis approach offers the capability to independently vary filling factor and size of the nanoparticles. Moreover, the application of alloy nanoparticles instead of pure Ag NPs allows controlling filament formation by limiting the amount of mobile silver species while simultaneously the nobler alloy component may act as a stable anchor in the matrix for enhanced reliability.

This work is devoted to the investigation of memristive switching at the level of individual alloy nanoparticles, which are embedded in a dielectric SiO_2_ matrix. In such nanoscale arrangements, the thorough experimental assessment of memristive switching renders rather challenging. Although there are a variety of reports on observations of filament formation by *in situ* TEM^28^, the high demands on sample preparation as well as the resulting severe changes in sample surface and the considerable effect of electron beam irradiation on the electrical properties of dielectric matrices impose certain restrictions to such TEM methods in the context of memristive devices^29^. In this work, we apply a facile, non-invasive nanoscale method to study the memristive action at the level of individual nanoparticles and visualize *in situ* any possible structural degradation. In this method we utilize a conducting atomic force microscope (C-AFM) operated in a mixed feedback loop to measure the nanoscale current-voltage (IV) characteristics of nanoparticle-based memristive devices against both structural and geometrical variations. By using a sharp PtIr tip (of radius 2 nm) as scanning electrode, the C-AFM method proved to be advantageous to test the local properties of nanoparticle-based memristive devices as it allows to perform measurements on only one nanoparticle at a time. To ensure this possibility, the contact force between the tip and the sample must be kept below 1.2 nN during electrical measurements to yield contact area of roughly 7 nm^2^ ^30^. Furthermore we report on the scalability of the nanoparticle-based approach towards multilayer nanocomposites and investigate limiting factors for device stability and reliability, such as the impact of adding an additional Cr contact layer.

In the following sections, we first (section 2.1) discuss nanoscale memristive switching with diffusive switching characteristics as observed utilizing C-AFM method at the level of individual noble metal alloy nanoparticles. Moreover, to achieve robust memristive action, devices must be subjected to a stress test in order to find the conditions under which failure occurs. Interestingly, C-AFM offers the possibility to monitor *in situ* signatures of plastic deformation and the subsequent device failure, which will be studied in terms of device stability and degradation against prolonged operations especially for devices with a Cr wetting layer in section 2.2. Finally, in section 2.3 the feasibility of expanding the concept of nanoparticle-based memristive devices to multi-stack nanocomposites will be covered and we will show that the transition towards multi-stack nanocomposites is a versatile approach to design robust memristive devices while preserving the diffusive switching characteristics of individual nanoparticles.

## Results and Discussion

**Figure 1:**
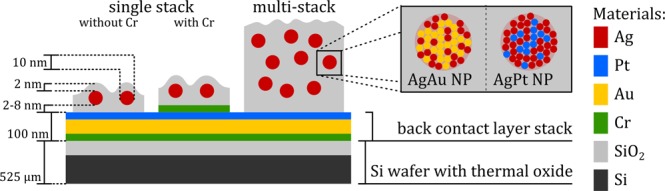
Schematic depiction of the typical setup of nanoparticle-based memristive devices in cross-sectional view, including approximate dimensions of the characteristic features. For a detailed assessment of memristive action of single nanoparticles, single stack SiO_2_/NP/SiO_2_ devices (with and without an additional Cr wetting layer) are characterized by C-AFM. The transition towards multi-stack arrangements allowed for reliable contacting of nanoparticle-based memristive devices with macroscopic electrodes.

In this work we investigate memristive switching relying on nanoparticle assemblies, which consist of noble metal alloy nanoparticles sandwiched between dielectric layers. The typical setup of individual SiO_2_/NP/SiO_2_ stacks as well as multi-stack nanoparticle-based devices is depicted schematically in Fig. 1.

As essential components, two types of noble metal alloy nanoparticles were investigated in the context of nanoparticle-based memristive switching, namely AgPt and AgAu nanoparticles. In both cases, the alloy nanoparticles exhibit a narrow size distribution with the mean diameter being roughly 11 nm (in case of AgAu) or 9 nm (in case of AgPt). TEM bright field micrographs of the respective AgAu and AgPt NPs are depicted in Fig. S1 in the supplementary data. The composition of the respective nanoparticles was determined by XPS (as depicted in Fig. S2 in the supplementary data) and the quantification yields a mole fraction of Ag of roughly 0.33 in case of AgAu nanoparticles and 0.73 in case of AgPt nanoparticles. More detailed investigations on AgAu nanoparticles deposited by an identical approach are shown in previous work^31^.

### Single nanoparticles for diffusive memristive switching

**Figure 2:**
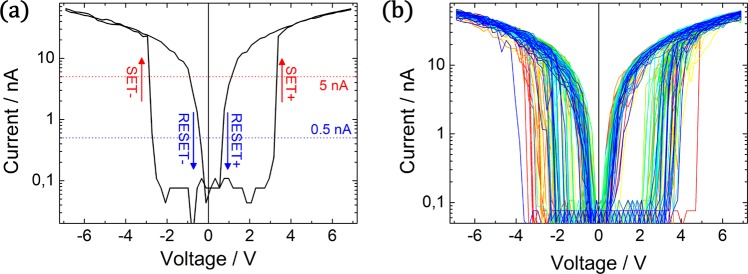
Diffusive memristive switching is observed by AFM measurements on an individual AgPt nanoparticle using a conductive tip. In a single hysteresis loop (**a**) the device shows a SET (switching towards LRS) and RESET (switching towards HRS) event for both, positive and negative polarity. For the reliable determination of the respective switching voltages, the current threshold of 5 nA is selected for a SET and 0.5 nA for a RESET. The comparison of 70 consecutive hysteresis loops (**b**) implies a certain distribution of the respective switching voltages. The individual hysteresis cycles are colour coded (first cycles: red colour, last cycles: blue colour).

Based on the aforementioned alloy nanoparticles, single SiO_2_/NP/SiO_2_ stacks were prepared and the electrical characteristics were recorded by C-AFM technique at the location of individual nanoparticles. The following discussion is devoted to the memristive switching properties of AgPt NPs in a SiO_2_/AgPt NP/SiO_2_ stack (nominal thickness of bottom and top oxide layer is 8 nm and 2 nm respectively), which is chosen as an example to illustrate the diffusive memristive switching behaviour observed in alloy NPs. The IV characteristics of this device are depicted in Fig. 2. A representative hysteresis loop of a full switching cycle is depicted in Fig. 2(a). Continuous measurements of 70 consecutive switching cycles are shown in Fig. 2(b).

The IV characteristics as depicted in Fig. 2(a) exhibit diffusive memristive switching (also termed threshold switching), thus for both polarities there is a transition from HRS to LRS (SET) and from LRS to HRS (RESET) and upon zero crossing, the device always is in its HRS. The full hysteresis loop of such nanoparticle-based memristive device can be described as follows:

In the initial state without bias, the nanoparticle device is in its HRS, which results in a current in the order of 100 pA, corresponding to the limit of detection. Upon increasing the voltage to a certain threshold, the device switches to its LRS and the IV curve is mainly dominated by the serial resistance of 101 MΩ (applied in order to limit the current through the C-AFM tip). When the applied voltage is subsequently reduced, the LRS is preserved until reaching a certain threshold voltage at which the device switches back into its HRS. A similar diffusive switching cycle with transitions from HRS to LRS and vice versa is observed at reversed polarity. In the following evaluation of the statistics of multiple cycles of diffusive memristive switching, the corresponding switching voltages are referred to as “SET+” and “RESET+” or “SET−” and “RESET−” for positive and negative polarity respectively.

In contrast to the diffusive memristive switching observed in individual alloy nanoparticles, the IV characteristics recorded on a pure SiO_2_ layer exhibits no indication for any switching event (cf. Fig. S6).

The origin of this diffusive memristive switching is expected to be related to the mechanism of electrochemical metallization (ECM)^32^. In the presence of an electrical field silver cations are released from their reservoir (in this case the individual nanoparticle), are transported through the SiO_2_ matrix and form a metallic filament upon being reduced. Due to the nanoscopic thickness of dielectric layer, even the limited amount of mobile silver species released from a single nanoparticle allows for the formation of a metallic filament, describing the transition from HRS to LRS above a certain threshold voltage.

For a thorough explanation of the RESET step, two aspects have to be considered. On the one hand, in case the nanoparticle-based device is in its LRS, a conductive filament is formed across the device and the potential drop is mainly over the serial resistor, which is applied to limit the current in the C-AFM measurement (cf. Fig. S9). The current flow through the metallic nanoparticle-based connection results in Joule heating and electromigration, which is typically associated with a RESET due to rupture of the metallic filament. On the other hand, the incorporation of alloy nanoparticles instead of bulk electrodes limits the amount of available silver species. Consequently, in our nanoparticle-based memristive devices the filament cannot grow to the full extent and is inherently unstable, which results in diffusive memristive switching. This is in contrast to conventional ECM devices relying on bulk electrodes, which offer an (almost) unlimited reservoir of mobile silver species and typically exhibit stable bipolar memristive switching.

Recently, Wang *et al*. reported a significant dependency of the filament lifetime on the diameter of the metallic filament, which is mainly motivated by the disintegration of a thin filament due to surface diffusion^33^. Similarly, our nanoparticle-based device can be put into the context of these considerations on filament lifetime by using the following assumptions: In case of a AgPt nanoparticle with a mole fraction of silver of roughly 0.73 and a diameter of 9 nm and a dielectric thickness of 8 nm would result in a filament diameter (under the assumption of a cylindrical filament) of less than 2.3 nm, which would result at zero bias, following the argumentation of Wang *et al*.^33^, in a filament lifetime of less than some tens of microseconds. Realistically, due to entropic considerations (entropy of mixing) it is unlikely that the whole amount of silver would be released from the respective nanoparticle and it is expected that the filament does not form in a perfectly cylindrical shape with constant diameter. Accordingly, the diameter and consequently the lifetime of the real filament are expected to be even lower, which immediately explains the observed diffusive switching and the instability of the LRS in the low voltage regime.

**Figure 3:**
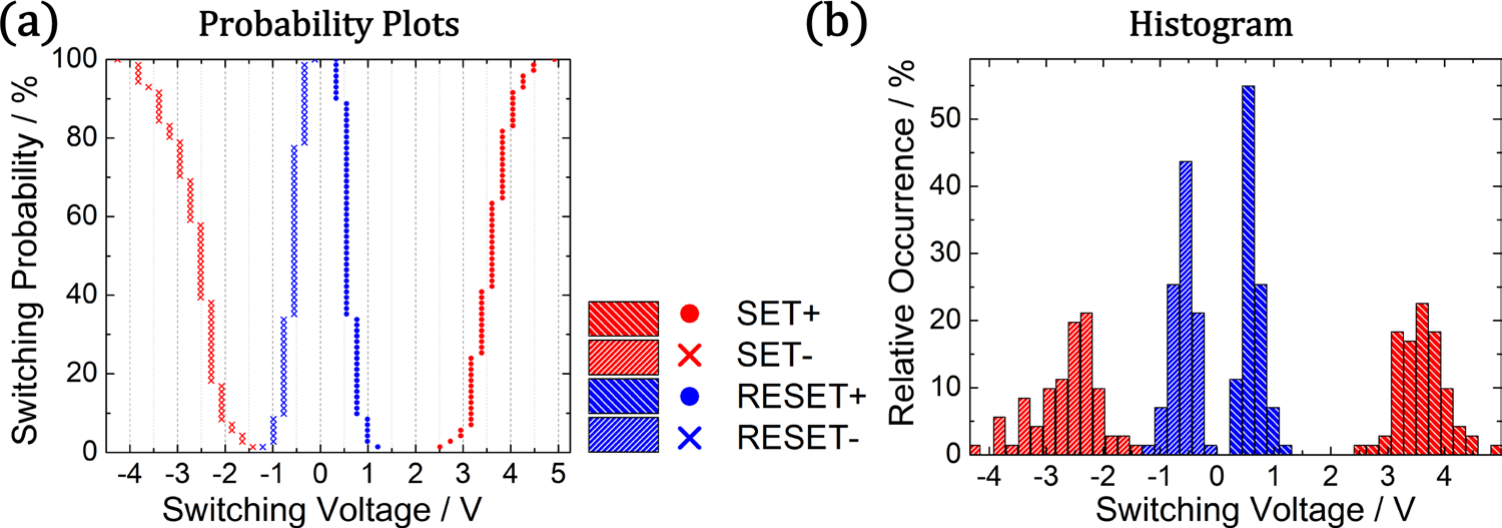
Statistical evaluation of the switching voltages obtained from 70 consecutive hysteresis loops measured for a SiO_2_/AgPt NP/SiO_2_ stack. The probability plot (**a**) and histogram (**b**) shows a general trend of higher SET voltages for positive polarity.

The IV characteristics were recorded at the location of an individual nanoparticle for 70 consecutive hysteresis cycles and are depicted in Fig. 2(b). In general, diffusive memristive switching is observed for each cycle and the respective switching voltages for the SET and RESET switching are distributed over a certain range. For a proper evaluation of the statistical distribution of switching voltages, the SET and RESET voltages are defined as the voltage at which the current raises above 5 nA or falls below 0.5 nA respectively. Based on these evaluation criteria, the switching voltages are extracted from the 70 hysteresis loops and are depicted by means of a cumulative switching probability plot (a) and a histogram (b) in Fig. 3.

In general, the SET+ voltage (3.60 ± 0.42 V) is shifted to higher voltages and exhibits a narrower distribution compared to the corresponding SET− process at opposite polarity (−2.65 ± 0.57 V). The observation of a higher SET+ voltage is in line with the expectation based on the asymmetry of the SiO_2_ separation layers (8 nm and 2 nm as bottom and top layer respectively). The histogram of the distribution of switching voltages implies that there is a clear separation between the SET and RESET voltages, especially at positive polarity. Within this operation window, both resistance states (LRS and HRS) are stable and the presence of a particular state depends on the history of applied voltage. Considering an interval of three standard deviations (1.26 V for SET+ and 0.54 V for RESET+) around the mean values (3.60 V and 0.62 V respectively), the corresponding operation window exhibits a width of 1.18 V with 99.7% confidence (under the assumption of a Gaussian distribution of the respective switching voltages).

**Figure 4:**
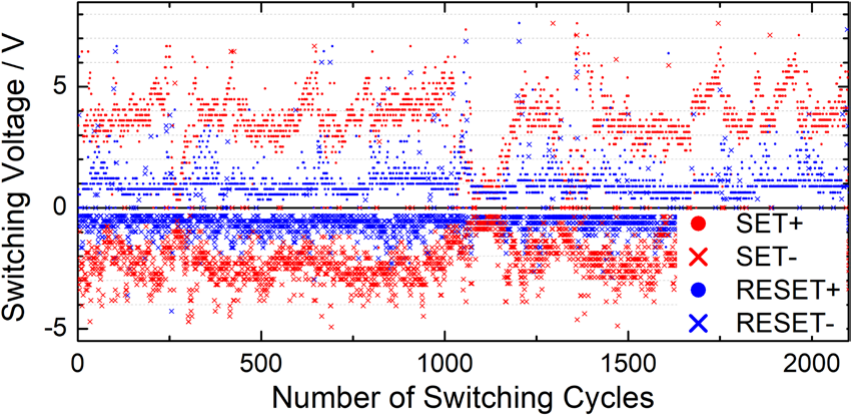
Overview over the switching voltages as extracted from individual hysteresis loops for 2000 switching cycles in a SiO_2_/AgPt/SiO_2_ stack, measured by C-AFM on an individual AgPt nanoparticle. While occasionally variations in the switching voltages occur due to limitations of the AFM setup (room temperature, ambient air), no systematic drift is observed.

Essentially, memristive switching on the basis of individual alloy nanoparticles was observed to be stable and reproducible switching behaviour was detected for many consecutive cycles by C-AFM method. For illustration, the switching voltages (as extracted from the individual hysteresis measurements) corresponding to 2000 consecutive cycles of a representative measurement on the SiO_2_/AgPt/SiO_2_ stack are depicted in Fig. 4. In general, the switching voltages are found to be statistically distributed as described in detail for 70 cycles in Fig. 3. In addition, for a small number of cycles (e.g. around cycle 1100), no memristive switching was observed and the detected switching voltages consequently turn out to be very low. A representative IV hysteresis loop for a cycle without distinct switching events is shown in Fig. S10. These occasional deviations in memristive behaviour may be attributed to the limitations of the C-AFM measurement, which is operated at ambient atmosphere and temperature. However, more importantly, no time-dependent, systematic drift of the switching voltages is observed, which indicates the high stability of memristive switching based on individual noble metal alloy nanoparticles.

Considering the insights gained by C-AFM measurements, nanoparticle-based memristive switching appears highly promising in the context of designing memristive devices with diffusive switching characteristics. For this purpose, two main design routes will be explored in the next sections, namely (1) the incorporation of a Cr wetting layer for a better conformity of the dielectric layer and (2) the transition towards multiple stacks of nanoparticles allowing for additional degrees of freedom for tailoring the switching parameters.

### Long-term stability of nanoparticle-based memristive switching in the presence of a Cr wetting layer

In thin film technology, transition metals such as Cr or Ti are widely applied to enhance the adhesion between different materials like SiO_2_ and Pt. In order to investigate the impact of the addition of a Cr layer on the electrical properties, a Cr/SiO_2_/AgPt/SiO_2_ thin film stack with SiO_2_ separation layer thickness of 2 nm was characterized by C-AFM. Over the whole measurement period of two days, IV hysteresis loops were recorded continuously and exhibited qualitatively similar behaviour (cf. Fig. S3 in supplementary data for a comparison of hysteresis loops recorded within the first hour and after two days).

**Figure 5:**
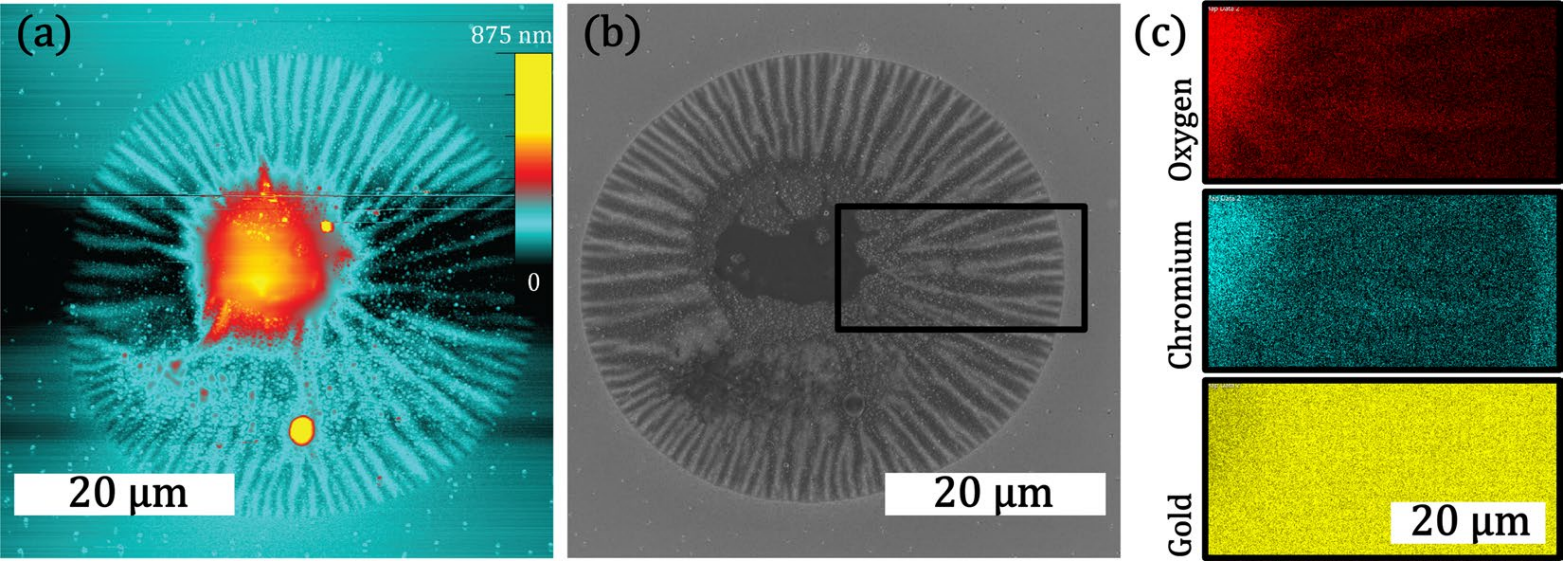
Morphological alterations of the thin film surface on the micrometre scale are induced by the migration of chromium during a two-day continuous IV-hysteresis measurement: The AFM topography map (**a**) as well as the corresponding SEM top view micrograph (**b**) indicate severe changes of the thin film morphology on a circular area with a diameter of roughly 40 µm. The migration and oxidation of Cr is revealed as the origin of these structural changes by SEM EDX spectroscopy maps (**c**) of a selected area (black rectangle).

While the electrical characteristics are preserved, severe morphological alterations of the nanoparticle thin film are observed. Initially, the surface is smooth and does not exhibit significant irregularities regarding roughness and topography. After the two-day measurement, a severe structural deformation is observed (Fig. 5), ranging with a radius in the order of 20 µm around the location of the tip. Within this area there are two dominating features: A dome-like elevation is located at the centre (red and yellow colour, diameter of roughly 20 µm) and radially surrounded by fluctuating height distributions (diameter of roughly 40 µm). Considering the nanoscopic size of the AFM tip, such morphological modifications on the microscale are particularly surprising.

In order to uncover the origin of these morphological changes, SEM and SEM EDX were applied to image and characterize the respective region. The overall appearance of the structural deformation as observed in AFM topography map is well reproduced in the SEM top view micrograph (Fig. 5(b)). A selected rectangular region, containing the dome-like structure (left) as well as the radial fluctuations (middle) and an undisturbed region (right) was investigated by SEM EDX. The occurrence of the elements oxygen, chromium and gold is depicted in terms of elemental maps in Fig. 5(c) (high colour saturation corresponds to high signal). While the dome-like structure shows strong signal corresponding to Cr and O and considerable signal corresponding to Au, the undisturbed region contains Cr and Au, but considerably less O. The presence of signal corresponding to Au is attributed to the conducting thin film stack on the substrate, which mainly consist of Au. Within the intermediate region, the stripe-like features correlate to a radial depletion of Cr. Within this region, the strong Cr and O signals overlap. Judging from the results of SEM EDX investigations, the morphological changes are attributed to the migration and oxidation of Cr, which is brought to the surface of the Cr/SiO_2_/AgPt/SiO_2_ thin film stack in the form of chromium oxide and results in the dome-like feature as well as the radial height fluctuations.

Notably, these morphological alterations evolve over time. While in the early stages (e.g. after one hour of continuous IV hysteresis measurement) already first indications of dome-like features are present (see AFM topography map in Fig. S3(a) in supplementary data), the radial features are growing at later stages. Alongside a steady change in the morphology, the electrical characteristics are qualitatively preserved over the whole measurement period of two days (as shown in Fig. S3(b,c) in Supplementary Data). Representative consecutive hysteresis loops for two thin film stacks with either AgPt or AgAu nanoparticles are depicted in Fig. S4 in the supplementary data. In contrast to similar stacks without Cr wetting layer, in these devices no diffusive memristive switching is observed within the investigated voltage range. The respective hysteresis loops remind of a typical cyclic voltammetry measurement and exhibit a non-zero crossing (no pinched hysteresis) as well as several peaks corresponding to oxidation and reduction processes. Although qualitatively the overall shape of the IV hysteresis loop remains similar for consecutive cycles, changes in peak position and height of the individual peaks are strong indications for instabilities and can be related to a change in active area due to the reported morphological alterations.

In essence, at the example of long-term measurement induced migration of Cr, C-AFM has proven as an efficient method to monitor device stability and degradation during prolonged operations. Due to the detected instability, the wetting layer of Cr is not considered to be feasible for the development of multi-stack memristive devices as described in following section.

### Memristive switching in multiple stacks of nanoparticles

The scalability of memristive switching devices is one key aspect concerning hardware implementation. The nanoscopic dimensions of the individual SiO_2_/NP/SiO_2_ layers (with the dielectric being only a few nanometres thick) make reliable contacting by conventional probes very challenging. Thus, we expand our investigation from single nanoparticles towards multi-stack devices, which consist of multiple stacks of nanoparticles embedded in a dielectric SiO_2_ matrix. The transition from individual layers towards multiple stacks results in an increase in overall device layer thickness and consequently reduces the risk of short circuiting by pin holes or due to mechanical failure (pinching). The multi-stack samples discussed in this section comprised of 5 layers of individual AgAu and AgPt nanoparticles separated by thin SiO_2_ layers in between. While in case of the AgAu NP multi-stack device the nominal layer thickness of the separating SiO_2_ layers was selected to be 2 nm, the AgPt NPs were nominally separated by 4 nm of SiO_2_. The different SiO_2_ separation layer thickness was chosen due to the difference in the silver concentration in the respective nanoparticles. Accordingly, for the design of a multi-stack device relying on nanoparticles two general degrees of freedom open up: On the one hand the composition of the respective alloy nanoparticles and on the other hand the width of the dielectric separation layer, which is deposited in between the deposition of the individual nanoparticle layers, can be tailored.

**Figure 6:**
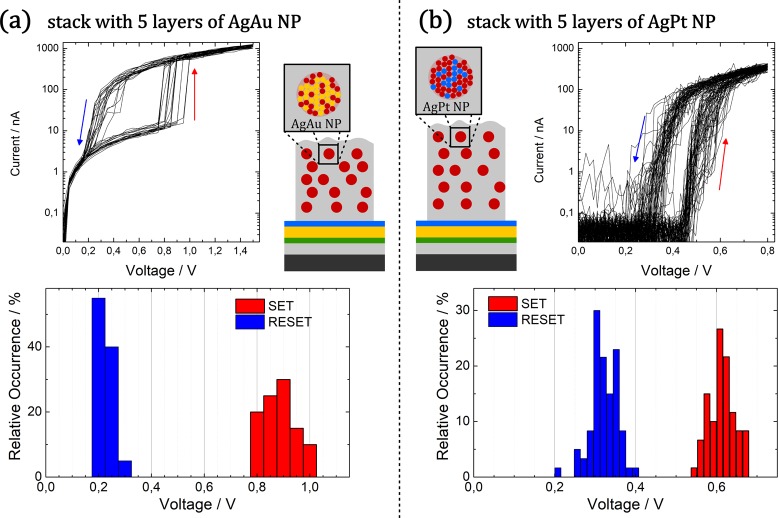
Multi-stack nanoparticle-based memristive devices relying on AgAu (left) and AgPt (right) nanoparticles exhibit diffusive memristive switching characteristics. (**a**) The switching characteristics are depicted for 20 consecutive cycles (top) of an AgAu nanoparticle device with 2 nm SiO_2_ separation layers. The corresponding histogram (bottom) shows a narrow distribution of SET (around 0.89 V) and RESET (around 0.23 V) voltages with a clear separation in between. (**b**) For a AgPt nanoparticle-based multi-stack device with SiO_2_ separation layers of 4 nm each, the switching characteristics are depicted for 60 consecutive cycles (top) and the corresponding histogram (bottom) shows a narrow distribution of SET (around 0.61 V) and RESET (around 0.32 V) voltages with a clear separation in between.

For the electrical characterization of the multi-stack devices, the top contact was realized by a soft Pt wire (combined with a serial resistance of 1 MΩ, see method section). As the Pt wire has a diameter of 125 µm, the effective contact is expected to be significantly larger than in case of the C-AFM investigations on single nanoparticles. Thus, the contact area expands from individual nanoparticles (in case of C-AFM measurements) towards a larger nanoparticle assembly. However, as shown in Fig. 6, the corresponding multi-stack devices featuring AgAu **(a)** and AgPt **(b)** nanoparticles show similar diffusive memristive switching, which demonstrates that upon upscaling from single nanoparticles to a multi-stack device the fundamental switching characteristics are preserved. Interestingly, no distinct electroforming step at higher voltages is required to initialize memristive switching in the nanoparticle-based devices, which underlines their application potential. For both, the AgAu and AgPt nanoparticle-based device, the IV hysteresis loops exhibit reproducible diffusive memristive switching over multiple consecutive cycles with a narrow distribution of the SET and RESET voltages, which underlines the fact that macroscopic contact hosts large number of independent nanoscale memristive devices. A closer look at the distribution of the switching voltages (cf. histograms in Fig. 6) indicates for both devices a distinct separation between the SET and RESET, which results in a stable operation window. The main differences between the AgAu and AgPt multi-stack device are found in the HRS resistance and the magnitude of the switching voltages.

While the AgAu device exhibits a HRS resistance in the order of 70 MΩ, a much higher resistance is observed for the AgPt device (current in HRS is below limit of reliable detection). This difference can be attributed to the SiO_2_ separation layer thickness, which is 2 nm in case of the AgAu device and 4 nm in case of the AgPt device.

While the switching voltages observed for the AgAu device are around 0.89 ± 0.06 V (SET) and 0.23 ± 0.03 V (RESET), the AgPt device exhibits a lower SET voltage around 0.61 ± 0.03 V and a higher RESET voltage around 0.32 ± 0.03 V. A comprehensive overview over the evaluated switching voltages is given in Table S1 in the supplementary data. Considering the higher SiO_2_ separation layer thickness in case of the AgPt device, the observed trend in the SET voltage at first glance seems rather counterintuitive, as the higher separation width is expected to result in a lower electrical field at identical applied voltage. However, the availability of Ag in case of AgPt nanoparticles (with Ag mole fraction of roughly 0.8) is much higher than in the AgAu device (with Ag mole fraction of roughly 0.3). Accordingly, the higher availability of silver species facilitates the filament formation (and as such the SET process).

In a similar approach, Wang *et al*. recently reported diffusive memristive switching in MgO_x_:Ag, SiO_x_N_y_:Ag and HfO_x_:Ag thin films, which were fabricated by co-sputtering in reactive atmosphere, and attributed the instability of the LRS to the coalescence of individual nanoparticles due to a minimization in surface energy^15^. The diffusive memristive switching characteristics of the multi-stack devices are very well competitive to these devices, especially with respect to the distribution of switching voltages as well as the operation window and switching stability. Unlike the multi-stack nanoparticle-based memristive devices, diffusive memristive devices prepared by Wang *et al*. incorporate Ag as mobile species in pure Ag nanoparticles, which are most likely formed by self-organization during co-sputtering. Following the concept of multi-stack nanoparticle-based memristive devices as reported in this work, the individual alloy nanoparticles as building blocks are already fully formed in the gas phase with a well-defined composition and size synthesis and are subsequently embedded into the dielectric, which grants multiple degrees of freedom (e.g. tailored filling factor and alloy composition) in the design of memristive devices.

## Conclusion

In this work nanoscale memristive switching is examined in memristive thin film devices, which rely on noble metal alloy nanoparticles of the system AgAu or AgPt that are embedded in a SiO_2_ dielectric matrix. Applying C-AFM as a non-invasive method and making use of the nanoscopic contact area of the tip, we studied the electrical characteristics at the level of individual nanoparticles and observed reliable memristive switching for several hours of continuous operations. In these nanoscale junctions, reliable diffusive memristive switching with a reasonably narrow distribution of SET and RESET voltages and clear operation window in between has been found. The observation of diffusive memristive switching implies that the formation of stable filaments and bipolar switching characteristics is efficiently suppressed by limiting the reservoir of potentially mobile silver species to the alloy nanoparticles. Besides the nanoscale electrical characterization, the method of C-AFM allows to monitor long term operation induced morphological alterations and device failure *in situ*, which is demonstrated at the example of nanoparticle-based devices with an additional Cr wetting layer. Herein, during continuous IV hysteresis measurement over a time period of two days, severe morphological alterations on the microscale originating from the migration and oxidation of Cr were detected by the C-AFM method.

In addition to the investigations on memristive action in individual alloy nanoparticles, the concept of nanoparticle-based memristive switching was extended to nanocomposites featuring assemblies of multiple stacks of nanoparticles. Notably, the diffusive memristive properties were found to be preserved in such multi-stack devices and the respective switching voltages exhibit a narrow distribution and a clear operation window. Accordingly, the underlying concept of embedding alloy nanoparticles as reservoirs for mobile metal cations in a dielectric matrix possesses a high versatility, which makes it highly promising for the future design of forming-free memristive switches with tailored diffusive switching properties.

## Methods

### Deposition of nanoparticle-based memristive devices

**Figure 7:**
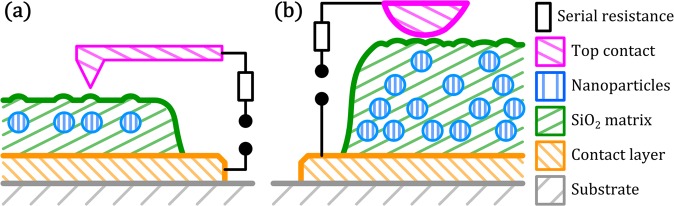
Schematic cross section of nanoparticle-based memristive devices for testing with C-AFM (**a**) or in a conventional two-probe setup (**b**). The nanoparticles (blue) are embedded in a dielectric matrix (SiO_2_, green), which is deposited on a metal contact layer (Au or Au/Cr, orange). The AFM tip (magenta) locally contacts individual nanoparticle switches, while macroscopic contacts such as a PtRh wire exhibit a significantly larger contact area.

The memristive devices consist of noble metal nanoparticles embedded into a dielectric SiO_2_ matrix. For the C-AFM investigations, a single layer of nanoparticles was deposited in between SiO_2_ layers (as schematically shown in Fig. 7(a)). In addition, stacks consisting of 5 layers of noble metal alloy nanoparticles separated by SiO_2_ were fabricated by consecutively depositing every layer (as schematically shown in Fig. 7(b) without breaking the vacuum. As substrates, phosphor doped, (100) oriented Si wafer pieces with native oxide (1 × 1 cm^2^, SiMat) were used. In order to achieve a smooth common back electrode, the substrates were coated with a stack of Cr, Au and Pt by magnetron sputtering (CS730S, Von Ardenne GmbH) from metallic Cr, Au and Pt targets respectively.

The fabrication of nanoparticle-based memristive devices was realized by physical vapour deposition (PVD) processes in a custom-build high vacuum (HV) deposition system. HV conditions were accomplished by using a turbo molecular pump (Pfeiffer Vacuum, TMU 262) combined with a dry scroll pump (Agilent Technologies, SH-110).

For the gas phase synthesis of noble metal nanoparticles, unipolar DC magnetron sputtering using an in-house Haberland type gas Aggregation Source (GAS)^34^ was applied. The GAS was separated from the main chamber by an orifice of 2 mm diameter at a distance of 90 mm from substrate position. The respective AgAu or AgPt target was attached to a DC planar magnetron source (Thin Film Consulting, ION’X-2UHV) inside the GAS. For the deposition of noble metal alloy AgAu and AgPt nanoparticles, a segmented target approach was employed^31^. The corresponding targets consist of Ag targets (Kurt J. Lesker, 99.99%, 5 cm diameter) with Au (Alfa Aesar, 1.0 mm dia, 99.95%) or Pt (Alfa Aesar, 1.0 mm dia, 99.95%) wires embedded in the racetrack. A flow of Ar (purity 99.999%) as process gas was regulated by gas regulating valve (Pfeiffer, EVR116 with attached hot ion cathode IMR 285) at the gas inlet of the GAS. DC power of 40 W was supplied by power source (Advanced Energy, MDX 500).

The dielectric SiO_2_ matrix was deposited by pulsed DC reactive magnetron sputtering either directly on the Pt surface of the substrate or on an intermediate Cr wetting layer. The Cr layer was deposited from a Cr target (Alfa Aesar, 99.95%, 2 inch diameter), attached to a magnetron (Thin Film Consulting, ION’X-2UHV) by DC magnetron sputtering (Advanced Energy, MDX 500) for 120 s at 100 W in a pure Ar plasma, which corresponds to a layer thickness of roughly 20 nm. For the deposition of SiO_2_, a Si target (Goodfellow GmbH, 99.999%, 2 inch diameter) was mounted onto a DC planar magnetron source (Thin Film Consulting, ION’X-2UHV) at a distance of 70 mm from substrate position. As a reactive gas, O_2_ (purity 99.999%) was introduced into the main chamber through a mass flow controller (Alicat, H-MC-100SCCM-D/5 M). Pulsing of the DC power source (Advanced Energy, MDX 500) was realized by a high power MOSFET (Behlke Power Electronics, HTS 31 CF I), which was triggered by a frequency generator (PeakTech, DDS Function Generator 4025, rectangular 0–5 V signal, 80 kHz, 30% duty cycle).

Prior to every deposition process the chamber was evacuated to 10^−4^ Pa and cleaning of target and conditioning of deposition were performed for sufficient time to reach stable deposition conditions, but at least for 30 s. Subsequently the shutter was opened and deposition performed.

### Electrical characterization of nanoparticle-based memristive devices

Electrical characterization of nanoparticle-based memristive switching was performed on the one hand by conductive atomic force microscopy (C-AFM) for the selective contacting of individual nanoparticles and on the other hand by a two-point measurement on a four-point probe station in case of the multi-stack devices.

C-AFM investigation was carried out using an AFM microscope (Dimension 3000, Bruker), which was modified to incorporate mixed feedback control as well as custom-made summing amplifier, resistor box and pulse generator for device initialization. The details of the measurements are described elsewhere^35^. Briefly, a topographic image was obtained using the conventional tapping mode technique, then the conducting AFM tip is positioned at the desired location before being lowered to contact the surface with a force of 1.2 nN. This force was kept constant during all measurements using a feedback system. A representative AFM topography map, clearly showing the sparse lateral distribution of nanoparticles, which is necessary for the contacting of individual nanoparticles, is provided in Fig. S5 in the supplementary data. The tip voltage was then ramped and the resulting current was detected using a fast preamplifier (Bruker C-AFM sensor). The shielded preamplifier’s input was closely positioned (about 25 mm away) from the cantilever’s contact to minimize noise in the low current measurements. The period of a typical IV cycle was typically 5 seconds with reading averaged 10 times at each point. To protect the thinly coated (20 nm thickness) PtIr AFM tip and the sample, especially during device initialization, the current was limited by a serial resistor (typically 1 GΩ or 101 MΩ). These settings proved to be non-invasive as we didn’t detect any changes to the topography or tip after the initialization process. To condition devices a few pulses of duration 30 ms at bias ≤5 V were applied to initialize ionic conduction within the memristive matrix. Once the current is detected, the voltage is ramped initially at a reduced range from −2 to 2 V, then increased gradually to higher values to capture the full range of switching onsets. In all cases the sweeping window is kept within ±7 V range with a maximum current of 70 nA. Most of the investigated devices have survived prolonged IV measurements when current is kept below 2 nA range, except those having Cr as a wetting layer.

The electrical characterization of the multi-stack nanoparticle devices was performed using a source meter (Keithley, 2400 Source Measure Unit) and a four-point probe station (Signatone, H150W). The common contact layer was contacted with a conventional tungsten tip (Signatone, SE-T) as a back electrode. In order to achieve a soft contact to the top of the memristive stack, a flexible PtRh wire (Alfa Aesar, 13 wt% Rh, 127 µm diameter) was used as a top contact. In analogy to the AFM setup, involving a serial resistance for all measurements, the PtRh wire was connected to a serial resistance in order to achieve comparable measurement conditions. The characterization by PtRh wire instead of conducting AFM tip allowed for higher currents, so that the serial resistance was chosen to be 1 MΩ. To measure the switching characteristics, DC voltage sweeps were applied with the top contact biased and the back electrode grounded. No current compliance was set by the source measure unit, as the serial resistance provides current limitation and suppresses overshoot effects. No electroforming steps at higher voltages were performed for measurements; instead memristive switching was initialized within the identical voltage range as applied for subsequently recorded IV hysteresis loops.

### Further characterization techniques

For a detailed analysis of the long-term measurement induced morphological changes in nanoparticle based memristive devices with additional Cr wetting layer, scanning electron microscopy (SEM; Zeiss, Supra 55VP) was performed in top view configuration. Chemical information was recorded by energy dispersive X-ray analysis (Oxford Instruments, x-act) in connection with the aforementioned SEM studies. For comparison, topographical information was obtained by AFM. AFM was performed in tapping mode with a rectangular cantilever (spring constant, k = 2 N/m) and tetrahedral tip, oscillated at its resonance frequency of 85 kHz. Areas of measurements were 50 × 50 µm^2^ and 3 × 3 µm^2^. Scanning area was divided by 512 lines per image and 512 points per line.

X-ray photoelectron spectroscopy (XPS, Omicron Full lab, Omicron Nano-Technology GmbH, Al-anode, 240 W, EA125 hemispherical analyser with pass energy 100 eV) was applied in order to determine the stoichiometry of the alloy nanoparticles of the system AgAu and AgPt, which were deposited onto a Si wafer piece with native oxide. During XPS analysis, the base pressure in the main chamber was of the order of 10^−7^ Pa. The C-1s line of advantageous carbon at 285.0 eV was used to correct the charging in all recorded spectra respectively by using the software CasaXPS (version 2.3.16).

Transmission electron microscopy (TEM) analysis was conducted using a FEI Tecnai F30 STwin microscope (300 kV, field emission gun (FEG) cathode, spherical aberration coefficient C_s_ = 1.2 mm). Micrographs of AgAu and AgPt nanoparticles on carbon film copper TEM grids (Plano, S160-4) were recorded in bright field mode.

### Data availability

The data generated and analysed in this study are shown in the present publication as well as the supplementary information and access to specific datasets is available from the corresponding author on reasonable request. This study was included in parts in the PhD thesis entitled “On the Development of Memsensors”^36^.

## Supplementary information

Supplementary Information
