# Supplementary material for: Diffusive Memristive Switching on the Nanoscale, from Individual Nanoparticles towards Scalable Nanocomposite Devices
Source: Sci Rep. 2019 Nov 22;9:17367. doi: 10.1038/s41598-019-53720-2 (PMC6874579; doi:10.1038/s41598-019-53720-2)
Supplement: Supplementary file 1 — Supplementary Information [file 41598_2019_53720_MOESM1_ESM.docx]

Supplementary Information

Diffusive Memristive Switching on the Nanoscale, from Individual Nanoparticles towards Scalable Nanocomposite Devices

*Alexander Vahl^1^, Niko Carstens^1^, Thomas Strunskus^1^, Franz Faupel^1*^, Abdou Hassanien^2*^*

^1^ Institute for Materials Science – Chair for Multicomponent Materials, Faculty of Engineering, Christian-Albrechts-University of Kiel, Kaiserstraße 2, D-24143 Kiel, Germany

^2^ Department of Condensed Matter Physics, J. Stefan Institute, Jamova 39, 1000 Ljubljana, Slovenia

*Correspondence to ff@tf.uni-kiel.de or Abdou.Hassanien@ijs.si

**Table S1**: Evaluation of diffusive memristive switching: Overview on the switching voltages extracted from multiple cycles (single nanoparticle AgPt device: 70 cycles; multi-stack AgAu device: 20 cycles; multi-stack AgPt device: 60 cycles). Mean (average) voltage: *V_mean_*; standard deviation: σ; voltage median: *V_med_*; minimum and maximum observed switching voltage: *V_min_* and *V_max_*.

| *Type of Memristive Device* | | *Switching Process* | *V_mean_* | *σ* | *V_med_* | *V_max_* | *V_min_* |
| --- | --- | --- | --- | --- | --- | --- | --- |
| single nanoparticle device | AgPt | SET+ / V | 3.60 | 0.42 | 3.61 | 4.92 | 2.51 |
|  |  | RESET+ / V | 0.62 | 0.18 | 0.55 | 1.21 | 0.33 |
|  |  | SET- / V | -2.65 | 0.57 | -2.51 | -4.26 | -1.42 |
|  |  | RESET- / V | -0.59 | 0.21 | -0.55 | -1.21 | -0.11 |
| multi-stack device | AgAu | SET / V | 0.89 | 0.06 | 0.90 | 1.00 | 0.80 |
|  |  | RESET / V | 0.23 | 0.03 | 0.20 | 0.30 | 0.20 |
|  | AgPt | SET / V | 0.61 | 0.03 | 0.61 | 0.67 | 0.54 |
|  |  | RESET / V | 0.32 | 0.03 | 0.32 | 0.4 | 0.21 |


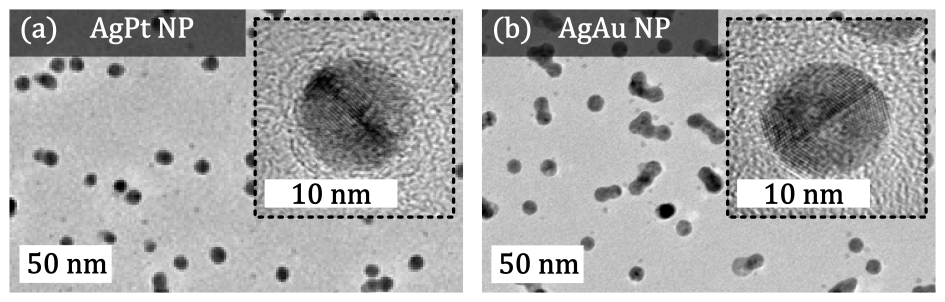


**Figure S1.** TEM bright field micrographs of AgPt (a) and AgAu (b) nanoparticles. The insets show a representative, individual nanoparticle at higher magnification. The mean diameter of the nanoparticles lies in the range of 10 nm (*r_mean,AgPt_* = 9 nm, *r_mean,AgAu_* = 11 nm).


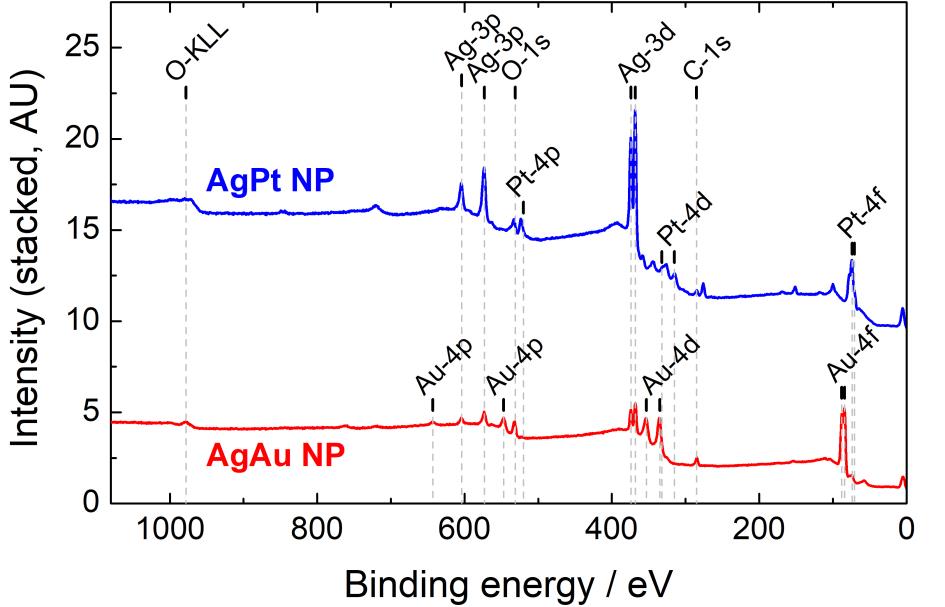


**Figure S2.** XPS spectra of AgPt (blue line) and AgAu (red line) nanoparticles deposited onto Si wafer pieces. The overview spectra reveal the presence of the elements Ag, Au, Pt, O and C.

For the quantification of the composition of the AgAu nanoparticles, the peak areas corresponding to the Ag-3d_5/2_, Ag-3d_5/2_ and Au-4d_5/2_ were taken into account, which yield a mole fraction of silver of roughly 0.33 and of gold of roughly 0.67. In case of the AgPt nanoparticles, the Ag-3p_3/2_ and Pt-4p_3/2_ lines were considered and the composition of the respective nanoparticles was determined to be roughly 0.73 Ag and 0.27 Pt in terms of mole fractions.


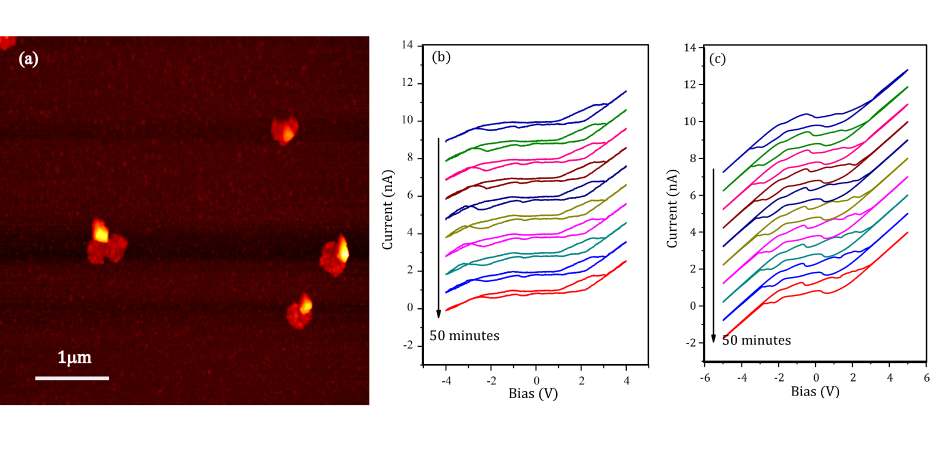


**Figure S3.** Early stages of nanoparticle-based memristive device operations by C-AFM measurements: (a) Morphological variations at four locations showing protrusions where IV hysteresis loops were taken within an hour of continuous operation. (b) Electrical measurements display stable IV characteristics during the first hour of operations. (c) Electrical measurements of the IV characteristics during the last hour after two days of continuous operation. The curves are offset for clarity.


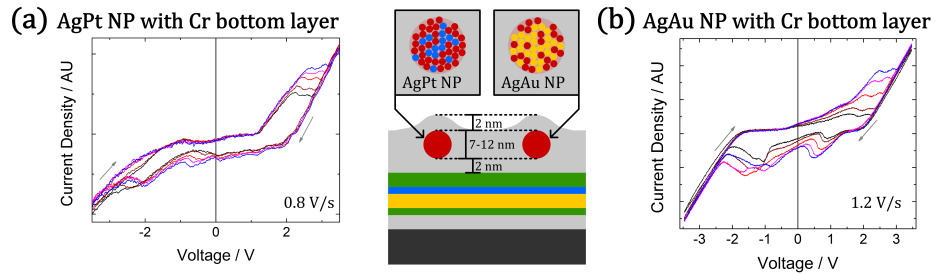


**Figure S4.** IV hysteresis measurements of nanoparticle-based memristive devices featuring AgPt (a) or AgAu (b) nanoparticles in the presence of an additional Cr wetting layer. While reproducible memristive switching with diffusive characteristics is observed for devices without Cr wetting layer, the hysteresis loops for Cr/SiO_2_/NP/SiO_2_ stacks show no indication of diffusive switching but peaks of varying position and height.

**
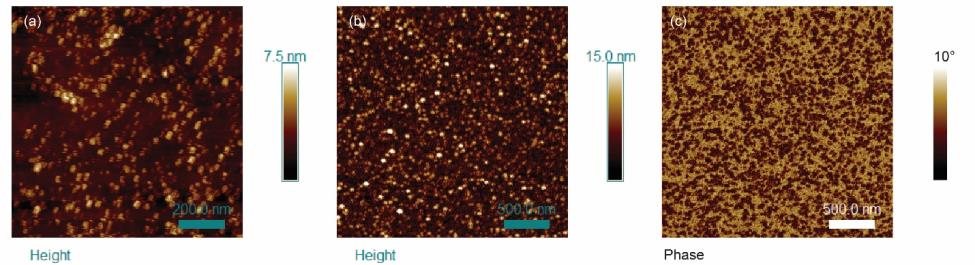
**

Figure S5. Topographic and Phase images of NP composites. (a) Individual AuAg NPs with an average height of 7 nm can be accessed by pointing the tip toward the desired positions. (b) Topographic image showing a NP layer with higher lateral density, which still can be accessed selectively by simultaneous mapping of the phase variation as shown in (c). The dark regions in (c) correspond to the locations of NPs.

**
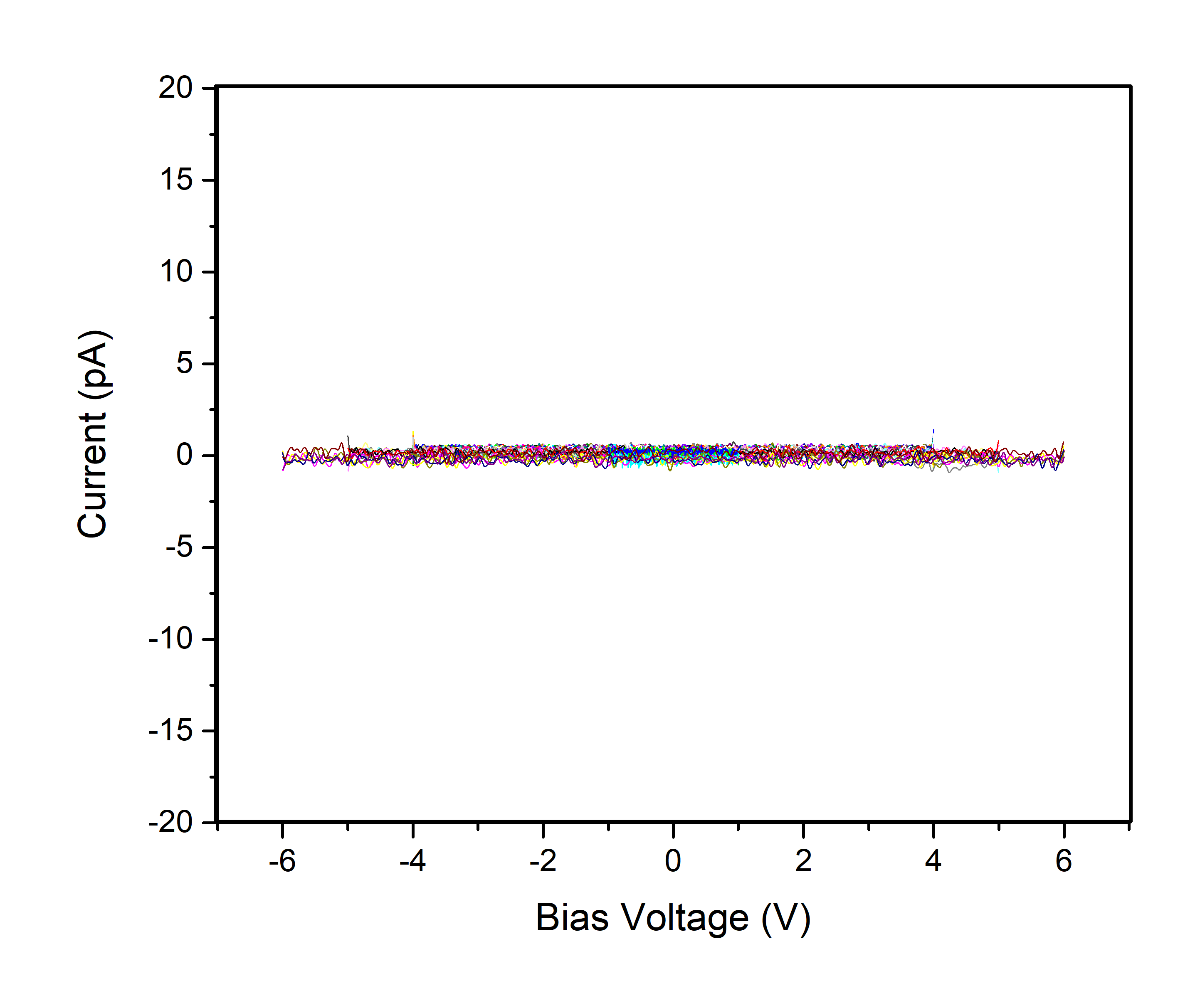
**

Figure S6. IV characteristics at various locations between NPs, i.e on empty SiO_2_ sites showing no detectable currents within the biasing window.

**
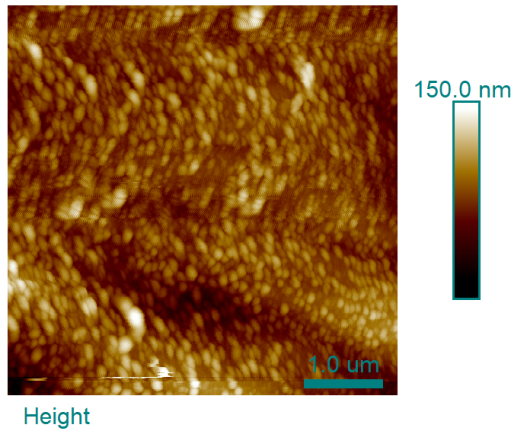
**

Figure S7. A close up view on the AFM topography map shown in Fig. 5, corresponding to the radial fluactuations in height.

**
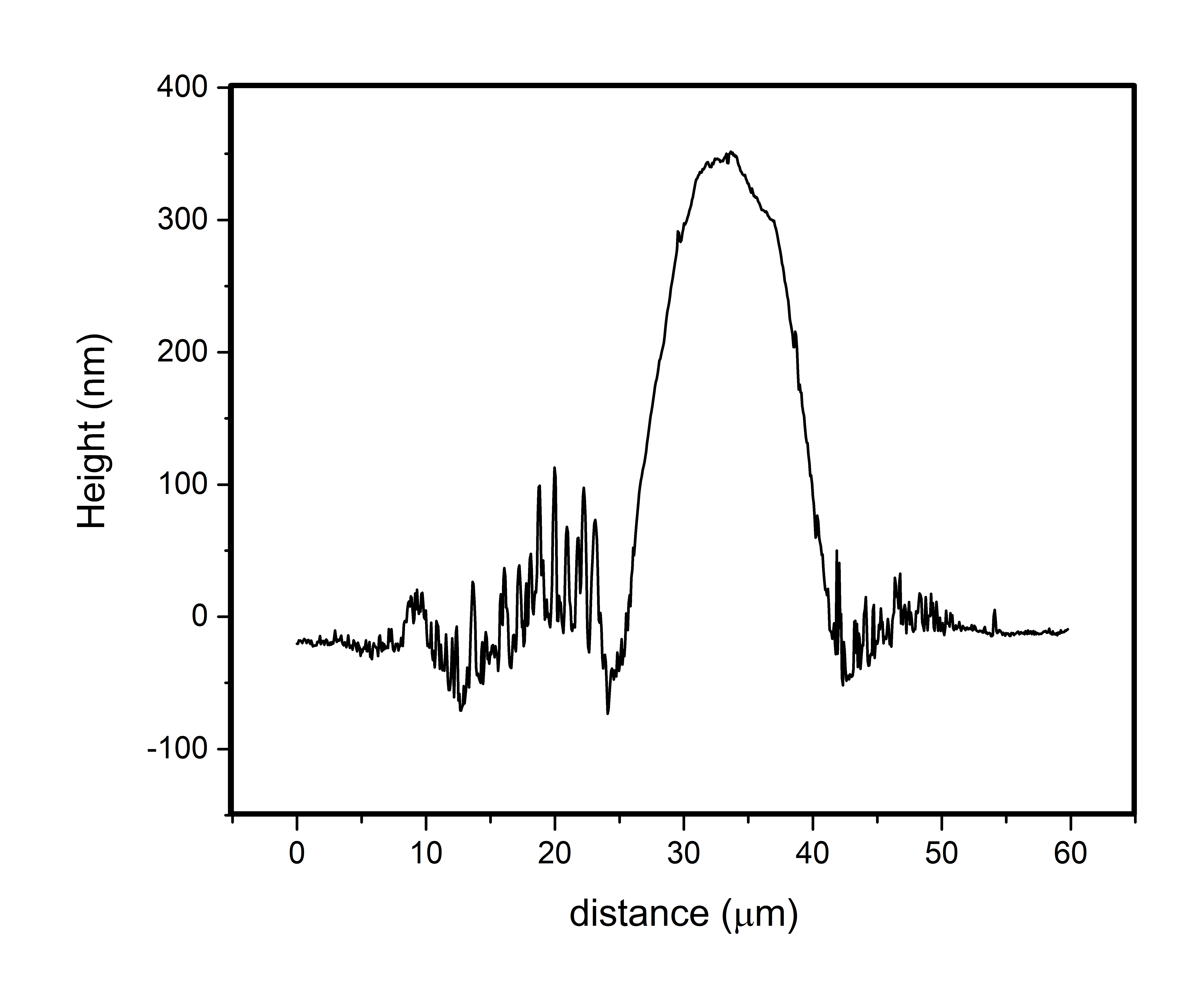
**

Figure S8. Line profile showing a dome like shape of the central region in a nanoparticle-based thin film stack with additional Cr layer. The amount of Cr within the diameter of the perturbed area (roughly 44 µm) is sufficient to feed the morphological variation of the central dome region.

**
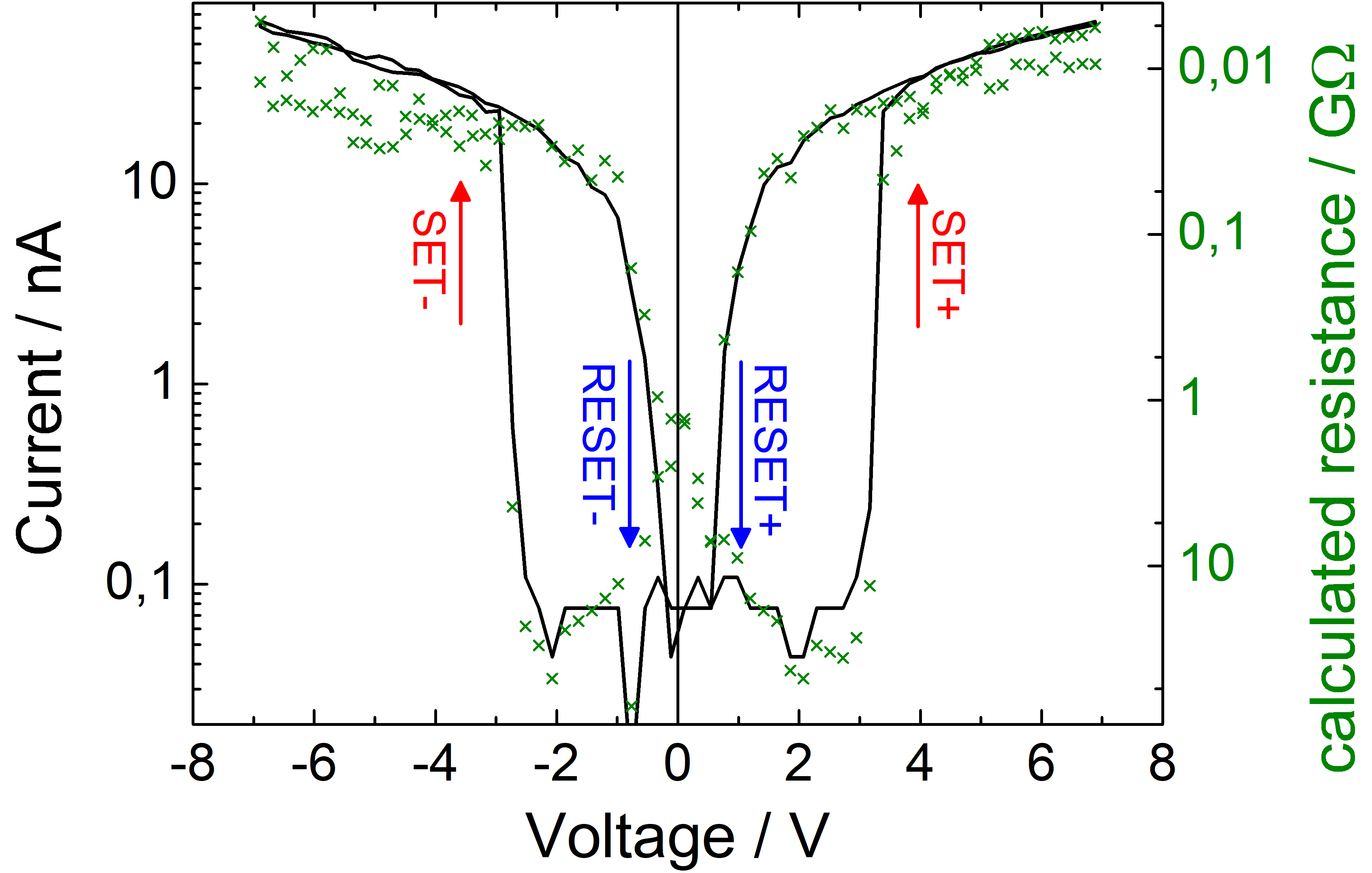
**

Figure S9. Diffusive memristive switching is observed in a representative IV hysteresis loop (black curve) measured by c-AFM on an individual AgPt nanoparticle. Considering a serial resistance of 101 MΩ, which was applied to protect the c-AFM tip from current overshoots, the calculated resistance of the diffusive filament is plotted in green.

**
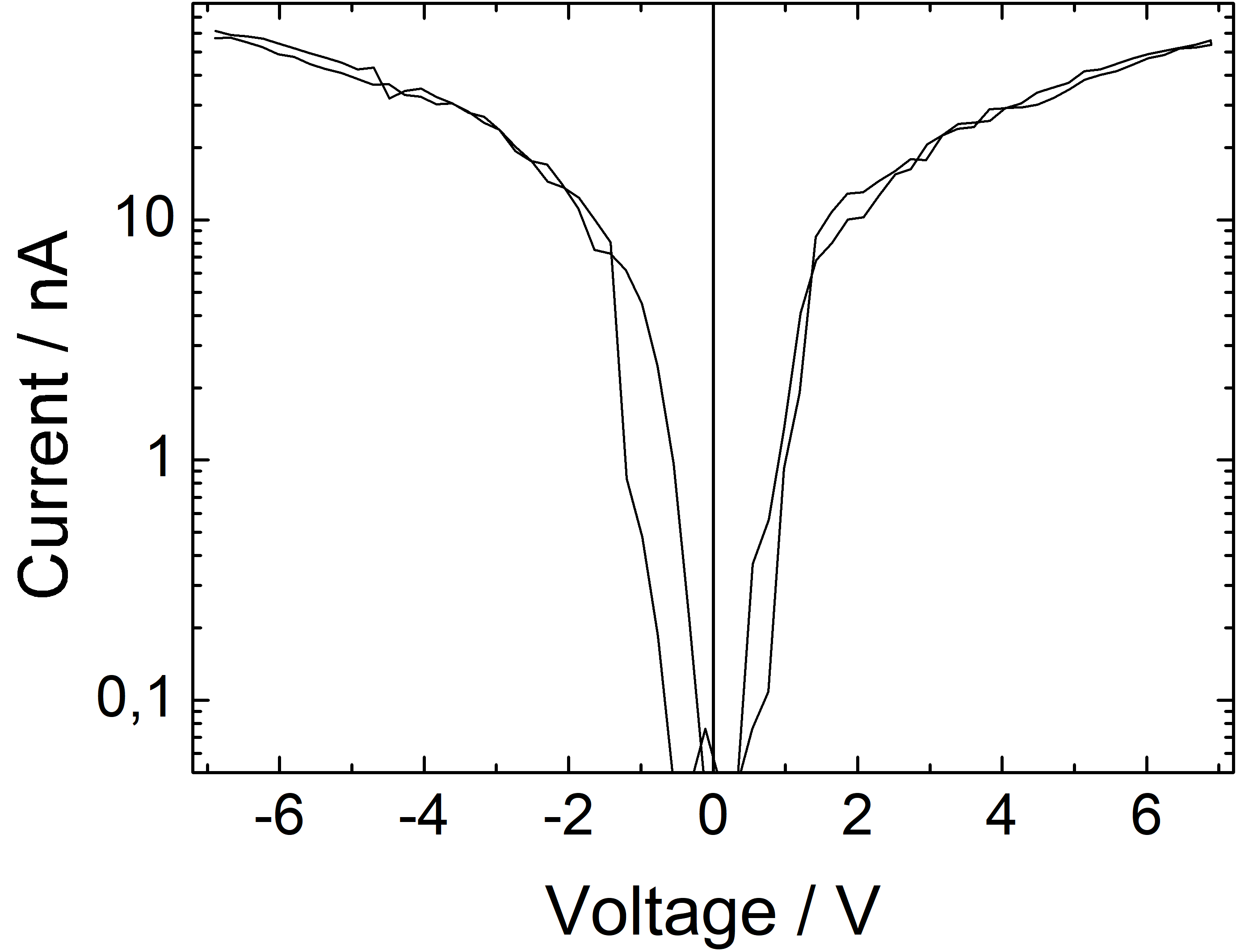
**

Figure S10. IV hysteresis loop obtained by c-AFM measurement on an individual AgPt nanoparticle for a representative cycle without distinct diffusive switching characteristics.

**
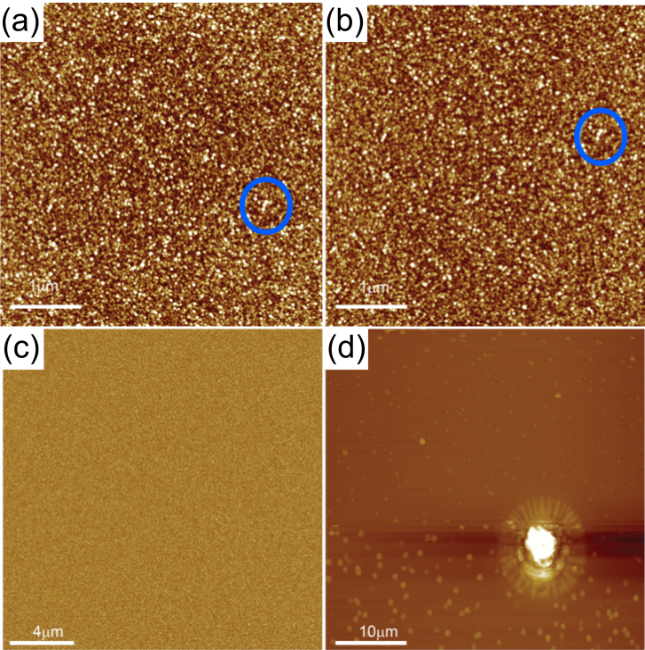
**

Figure S11. Long-term measurement induced morphological changes: While in case of a thin film stack without Cr (a-c) no morphological variations are observed, the addition of Cr leads to severe morphological alterrations (d). The topography of a AgAu NP device without Cr is identical before (a) and after (b) the electrical measurements at the location marked by the blue overlays. Also on a larger view AFM topography map of the respective region after measurements no detectable morphological variation occurs. (d) For a NP thin film stack with an additional Cr layer, dramatic changes in the topography are observed. These changes depend on the duration of the measurements, i.e small protrusions are due to few cycles of IV measurements, while the large feature in the lower right is due to a continous measurement (10 hours).
